# Activity profiling of barley vacuolar processing enzymes provides new insights into the plant and cyst nematode interaction

**DOI:** 10.1111/mpp.12878

**Published:** 2019-10-12

**Authors:** Mateusz Labudda, Elżbieta Różańska, Beata Prabucka, Ewa Muszyńska, Dorota Marecka, Marcin Kozak, Abdelfattah A. Dababat, Mirosław Sobczak

**Affiliations:** ^1^ Department of Biochemistry and Microbiology, Institute of Biology Warsaw University of Life Sciences‐SGGW Warsaw Poland; ^2^ Department of Botany, Institute of Biology Warsaw University of Life Sciences‐SGGW Warsaw Poland; ^3^ International Maize and Wheat Improvement Center (CIMMYT) Soil Borne Pathogens Program P.K. 39 Emek 06511 Ankara Turkey

**Keywords:** cyclotide, cystatin, *Heterodera filipjevi*, *Hordeum vulgare*, kalata B1, legumain, methuosis

## Abstract

Vacuolar processing enzymes (VPEs) play an important role during regular growth and development and defence responses. Despite substantial attempts to understand the molecular basis of plant–cyst nematode interaction, the mechanism of VPEs functioning during this interaction remains unknown. The second‐stage *Heterodera filipjevi* juvenile penetrates host roots and induces the formation of a permanent feeding site called a syncytium. To investigate whether infection with *H. filipjevi* alters plant host VPEs, the studies were performed in *Hordeum vulgare* roots and leaves on the day of inoculation and at 7, 14 and 21 days post‐inoculation (dpi). Implementing molecular, biochemical and microscopic methods we identified reasons for modulation of barley VPE activity during interaction with *H. filipjevi*. *Heterodera filipjevi* parasitism caused a general decrease of VPE activity in infected roots, but live imaging of VPEs showed that their activity is up‐regulated in syncytia at 7 and 14 dpi and down‐regulated at 21 dpi. These findings were accompanied by tissue‐specific VPE gene expression patterns. Expression of the barley cystatin *HvCPI‐4* gene was stimulated in leaves but diminished in roots upon infestation. External application of cyclotides that can be produced naturally by VPEs elicits in pre‐parasitic juveniles vesiculation of their body, enhanced formation of granules, induction of exploratory behaviour (stylet thrusts and head movements), production of reactive oxygen species (ROS) and final death by methuosis. Taken together, down‐regulation of VPE activity through nematode effectors promotes the nematode invasion rates and leads to avoidance of the induction of the plant proteolytic response and death of the invading juveniles.

## Introduction

Barley (*Hordeum vulgare*), one of the oldest domesticated cereals (over 10 millennia old), is known to be a crop species well‐adapted to changing and stressful environments and thus may be successfully cultivated even under adverse conditions (Dawson *et al.*, 2015; Kharub *et al.*, 2017). It is the fourth most important cereal in the world after maize, rice and wheat. Its grains are a valuable forage for livestock and food for humans, and their biochemical properties are used in malting and brewing (Dababat and Fourie, 2018; Kharub *et al.*, 2017). Barley is also one of the best‐studied cereals in terms of biochemistry, physiology, genetics, omics and molecular breeding, therefore it is considered to be an excellent model for cereals (Saisho and Takeda, 2011; Sreenivasulu *et al.*, 2008). Despite barley’s tolerance to stressful environmental conditions, pathogens can diminish yield and quality of grains, and thus barley genetic potential cannot be fully expressed (Dawson *et al.*, 2015). Among the pathogens that have a jeopardizing effect on crop plants, cyst nematodes are among the most significant (Labudda, 2018).

Cereal cyst nematodes (CCN) are a group of closely related species that parasitize grasses. Currently, 11 CCN species have been identified. Of these *Heterodera avenae*, *Heterodera latipons* and *Heterodera filipjevi* are considered the most economically important for global cereal production (Toumi *et al.*, 2018). The rye cyst nematode (*H. filipjevi*), like other CCNs, is an obligate biotrophic sedentary endoparasite, mainly infesting barley and wheat (Dababat and Fourie, 2018). As a result of *H. filipjevi* feeding on roots of infected plants the grain yield losses may reach up to 50% (Pariyar *et al.*, 2016). The infective second‐stage juvenile (J2) of CCN damages the host root cell wall using its stylet and hydrolytic enzymes synthesized in oesophageal glands and secreted through the stylet orifice. Subsequently the J2 migrates intracellularly within the root cortex and induces development of a multicellular syncytium in the vascular cylinder, which is the only the source of nutrients for the developing nematode during its sedentary life phase (Bohlmann and Sobczak, 2014; Toumi *et al.*, 2018). Nematode feeding inside the roots causes dramatic reorganization of the metabolism in all plant organs. Induced changes encompass activation of local (in roots) and systemic (in shoots) defence responses, among which proteolysis is an extremely important housekeeping process that must be mentioned (Labudda *et al.*, 2016a,b, 2018).

Proteolysis is an essential process in plants that employs different types of proteolytic enzymes, mainly serine (http://www.chem.qmul.ac.uk/iubmb/enzyme/EC3/4/21.html), aspartic (http://www.chem.qmul.ac.uk/iubmb/enzyme/EC3/4/23.html), metallo‐ (http://www.chem.qmul.ac.uk/iubmb/enzyme/EC3/4/24.html) and cysteine (http://www.chem.qmul.ac.uk/iubmb/enzyme/EC3/4/22.html) peptidases. Cysteine peptidases have been recognized as one of the crucial players in plant–nematode molecular interactions since both plants and nematodes use them to digest foreign proteins during the infection progress (Shindo and van der Hoorn, 2008). Cysteine peptidases are divided in the MEROPS peptidase database (https://www.ebi.ac.uk/merops/) into over a dozen clans. Plant vacuolar processing enzymes (VPEs), also called asparaginyl endopeptidases or legumains, are classified as members of clan CD and family C13 (http://www.chem.qmul.ac.uk/iubmb/enzyme/EC3/4/22/34.html) (Dall and Brandstetter, 2016). VPEs have many features in common with animal caspases. They are cysteine peptidases with a catalytic dyad occurring in the order histidine–cysteine. They are produced as inactive zymogens, break down peptide bonds at the C‐terminal sides after aspartic acid and asparagine residues, and are inactivated by similar inhibitors (Hatsugai *et al.*, 2015; Rantong and Gunawardena, 2017). However, VPEs are mainly accumulated in vacuoles, whereas animal caspases are present in the cytosol. As plants do not have macrophages, the dying plant cell hydrolyses its proteins by itself and VPEs play a key role in the regulation of the plant vacuole‐dependent proteolytic system during growth and development and defence responses against pathogens (Hara‐Nishimura *et al.*, 2005). Furthermore, VPEs participate in maturation and activation of vacuolar proteins and, as self‐activating peptidases, they do not require any other factor to acquire catalytic properties. Thus, VPEs are considered as initiators of the vacuolar protein processing machinery (Hatsugai *et al.*, 2015). Moreover, recently it has been shown that the organization of the vacuolar system in the syncytium induced by the beet cyst nematode *H. schachtii* in *Arabidopsis thaliana* roots changes dynamically during syncytium development, which indicates the importance of this cell compartment during plant–nematode interactions (Baranowski *et al.*, 2018).

The activity of proteolytic enzymes must be strictly controlled by their endogenous inhibitors. Phytocystatins are known to be one of the protein inhibitors of cysteine peptidases from the papain subfamily C1A. However, some phytocystatins have a carboxy‐terminal extension; thus they have also the ability to bind to legumains (family C13) and to inhibit their enzymatic activity. Interestingly, only one cystatin, HvCPI‐4, encoded by the *Icy‐4* gene, has been identified in barley so far (Velasco‐Arroyo *et al.*, 2018).

Our understanding of plant legumain biochemistry has expanded dramatically in recent years. Particular attention has been devoted to the elucidation of the role of VPEs in seed developmental processes (Julián *et al.*, 2013; Nakaune *et al.*, 2005; Radchuk *et al.*, 2011, 2018), but also there are several reports suggesting their involvement in plant responses to pathogens such as tobacco mosaic virus (Hatsugai *et al.*, 2004), *Erwinia amylovora* (Iakimova *et al.*, 2013), *Hyaloperonospora arabidopsidis* (Misas‐Villamil *et al.*, 2013), *Botrytis cinerea* (Rojo *et al.*, 2004), *Moniliophthora perniciosa* (Santana *et al.*, 2016) and *Cladosporium fulvum* (Sueldo *et al.*, 2014), and the cyst nematode *Heterodera schachtii* (Hütten *et al.*, 2015).

Furthermore, VPEs can also show ligase activity and they participate in the synthesis of cyclotides, cyclic disulphide‐rich peptides containing a head‐to‐tail cyclic backbone and a cystine knot (Dall and Brandstetter, 2016; Huang *et al.*, 2009). These unique peptides exert strong toxic effects on larval and adult individuals of sheep gastrointestinal nematodes *Haemonchus contortus* and *Trichostrongylus colubriformis* (Colgrave *et al.*, 2008, 2010), canine and human hookworms *Ancylostoma caninum* and *Necator americanus* (Colgrave *et al.*, 2009), and human digenean schistosomes *Schistosoma mansoni* and *Schistosoma japonicum* (Malagón *et al.*, 2013). One of the known mechanisms of cyclotide toxicity is the formation of multimeric pores in membranes (Huang *et al.*, 2009). The interaction of cyclotide kalata B1, the prototypic cyclotide from the African plant *Oldenlandia affinis*, with epithelial cells in the midgut of lepidopteran larvae of *Helicoverpa armigera* is a classic example of the negative impact of cyclotides on plant pests. Kalata B1 leads to inhibition of growth of *H. armigera* larvae, damage to the microvilli, blebbing, swelling and finally rupture of the gut epithelium cells (Barbeta *et al.*, 2008). This issue is particularly interesting since, to our knowledge, there is no available literature concerning the possible effects of cyclotides on plant‐parasitic nematodes.

Parasitism of cyst nematodes on host roots causes profound local metabolic changes in infested roots as well as systemic responses in leaves. To learn more about the molecular basis of plant–nematode interactions, we concentrated on the gene expression and enzyme activity of barley VPEs. The physiological role of VPEs in cereal plant responses to cyst nematode infestation is unknown and it has never been examined before. We aimed to explain the mechanism of VPE action on the molecular battlefield between the host plant and the invading nematode. Based on recently published data suggesting decreased protease activity in *H. schachtii*‐infected *A. thaliana* (Labudda *et al.*, 2016b), we hypothesized that VPEs could be important players in the barley response to *H. filipjevi* parasitism. Consequently, the main objective of the present study was to examine whether, to what extent and for what purpose the activity of barley VPEs is modulated during *H. filipjevi* infection. To solve these issues, molecular, biochemical and enzymological as well as confocal laser scanning and light microscopy methods were implemented.

## Results

### Chlorophyll content alteration

The concentrations and composition of photosynthetic pigments were measured over the entire examination period to test the physiological status of the infected plants. The analyses revealed the following photosynthetic pigment contents in *H. vulgare* specimens at 0 days post‐inoculation (dpi): 1.082 mg/g FW for chlorophyll *a* (chl *a*)*,* 0.255 mg/g FW for chlorophyll *b* (chl *b*) and 0.262 mg/g FW for carotenoids (Table 1). During the course of experiment, a slight decrease in pigment concentration was found in infected plants as compared to uninfected control; nevertheless the differences were statistically insignificant at 7 and 14 dpi. The nematode infection contributed to significant decrease of both chlorophyll concentrations only at 21 dpi, but the carotenoid content still did not differ between control and infected plants. Chlorophyll *a*:chlorophyll *b* ratio and chlorophyll *a* + *b* were almost 1.3‐ and 1.7‐fold lower, respectively, in infected than in uninfected plants at this time point. Furthermore, the reduction of chlorophyll *a* + *b*:carotenoids ratio occurred in infected specimens compared to uninfected ones (Table 1).

**Table 1:** Photosynthetic pigment content and its ratio in leaves of *Hordeum vulgare* on infection with *Heterodera filipjevi* at 7, 14 and 21 days post‐inoculation (dpi).

Parameter	Treatment*	7 dpi	14 dpi	21 dpi
Chlorophyll *a* (mg/g FW)	C	0.919 ± 0.041 a	0.638 ± 0.048 a	0.809 ± 0.175 a
	I	0.732 ± 0.132 a	0.574 ± 0.191 a	0.506 ± 0.009 b
Chlorophyll *b* (mg/g FW)	C	0.209 ± 0.009 a	0.138 ± 0.011 a	0.169 ± 0.034 a
	I	0.167 ± 0.030 a	0.125 ± 0.034 a	0.081 ± 0.011 b
Chlorophyll *a*:chhlorophyll *b*	C	4.397 ± 0.034 a	4.614 ± 0.050 a	6.351 ± 0.068 a
	I	4.394 ± 0.030 a	4.534 ± 0.534 a	4.761 ± 0.965 b
Chlorophyll *a* + *b* (mg/g FW)	C	1.128 ± 0.051 a	0.776 ± 0.059 a	0.978 ± 0.209 a
	I	0.899 ± 0.162 a	0.698 ± 0.224 a	0.587 ± 0.002 b
Carotenoids (mg/g FW)	C	0.239 ± 0.002 a	0.205 ± 0.016 a	0.228 ± 0.053 a
	I	0.214 ± 0.025 a	0.174 ± 0.065 a	0.173 ± 0.006 a
Chlorophyll *a* + *b*:carotenoids	C	4.714 ± 0.206 a	4.082 ± 0.071 a	4.303 ± 0.088 a
	I	4.189 ± 0.293 a	3.794 ± 0.335 a	3.394 ± 0.133 b

* C, leaves of control plants; I, leaves of nematode‐infected plants.

Values are means of three replicates ± SD. Different letters and grey background indicate statistically significant differences between treatment within each parameter and the period of experiment at α = 0.05.

### *Heterodera filipjevi* infection diminishes total legumain activity in infected roots

**Figure 1:**
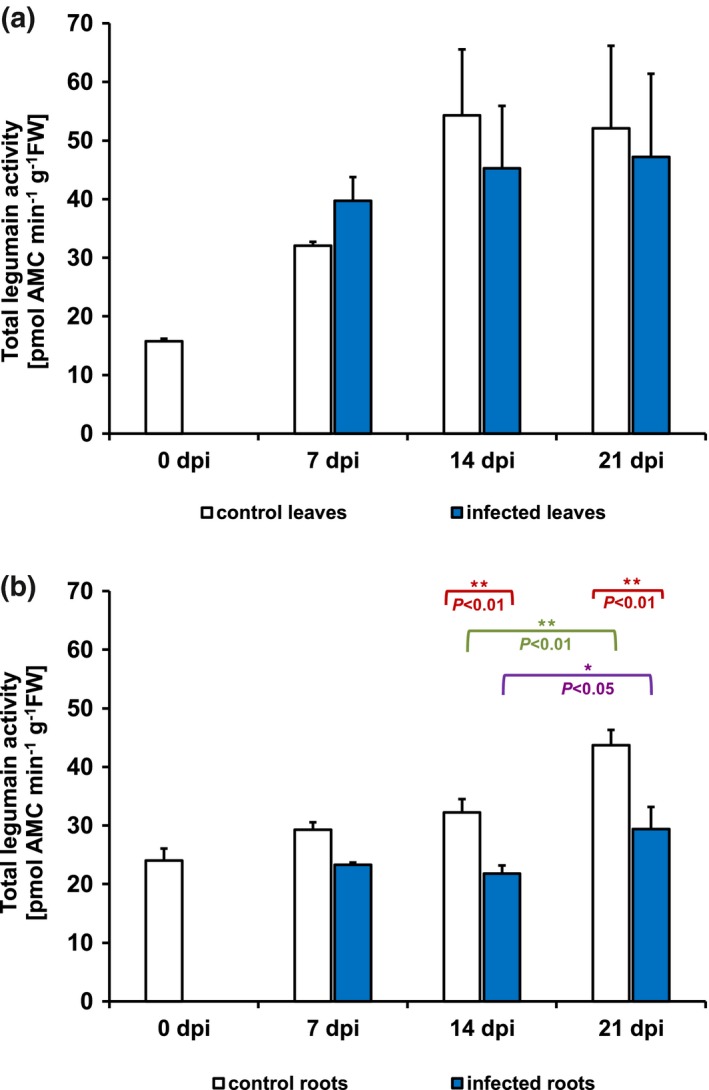
Total legumain activity (pmol AMC/min/g FW) in *Hordeum vulgare* leaves (a) and roots (b) on infestation with *Heterodera filipjevi* at 0, 7, 14 and 21 days post‐inoculation (dpi). Each result is presented as the mean ± SD (*n* = 3); significant difference at * *P < *0.05 or ** *P* < 0.01 (Tukey’s test). AMC, 7‐amino‐4‐methylcoumarin; FW, fresh weight.

To investigate VPE activity during *H. vulgare*–*H. filipjevi* interaction, the total legumain activity (TLA) was measured. On the day of inoculation, the TLA was lower in leaves (15.7 pmol 7‐amino‐4‐methylcoumarin (AMC)/min/g FW) than in roots (24 pmol AMC/min/g FW) (Fig. 1). At 7 dpi, the TLA was insignificantly lower in the roots of infected barley than in control roots (Fig. 1b), but it was significantly reduced by about 1.5‐fold in the infected roots at 14 and 21 dpi (Fig. 1b). In contrast, the TLA in the leaves of infected barley did not show significant differences throughout the experiment. Consequently, the investigation of VPE gene transcript levels was performed to establish whether altered TLA in infected plants coexists with changes in gene expression.

### Changes in gene expression of VPEs are organ‐specific

**Figure 2:**
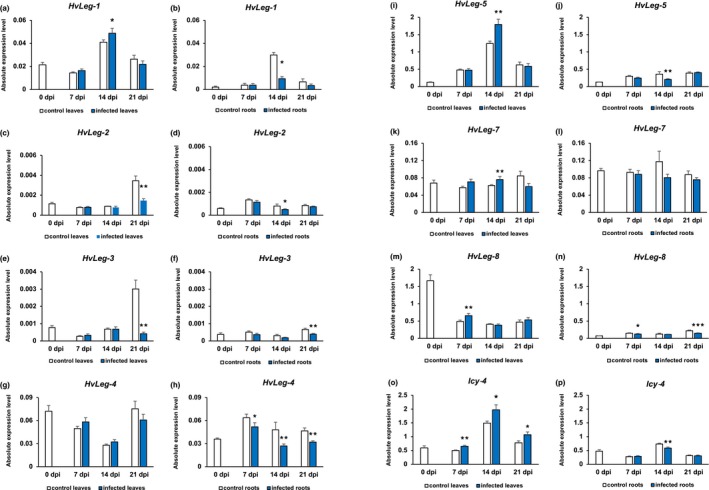
The analysis of changes in *Hordeum vulgare* vacuolar processing enzymes (*HvLeg*) (a–n) and cystatin HvCPI‐4 (*Icy‐4*) (o–p) gene expression levels during *Heterodera filipjevi* infection. Statistical analysis was carried out using REST2009 software (calculation of absolute normalized gene expression level and statistical significance of their differences). The expression level for each gene was normalized to the endogenous control. Bars represent mean ± SE (*n* = 3); asterisks indicate statistically significant differences in comparison to the control uninfected plants at *P* < 0.05 (*), *P* < 0.01 (**) or *P* < 0.001 (***).

qRT‐PCR analyses distinguished different gene expression patterns of VPEs in leaves (Fig. 2a,c,e,g,i,k,m) and roots (Fig. 2b,d,f,h,j,l,n) of infected barley. The statistically significant down‐regulation of the expression in infected roots concerned *HvLeg‐1* at 14 dpi (Fig. 2b), *HvLeg‐2* at 7 dpi (Fig. 2d), *HvLeg‐3* at 21 dpi (Fig. 2f), *HvLeg‐4* at 7, 14 and 21 dpi (Fig. 2h), *HvLeg‐5* at 14 dpi (Fig. 2j), and *HvLeg‐8* at 7 and 21 dpi (Fig. 2n). The dynamics of these changes varied depending on the time after inoculation. Only *HvLeg‐4* mRNA level was lower in infected roots in comparison with uninfected ones throughout the entire experiment (from 7 to 21 dpi) (Fig. 2h). In leaves of infected plants, transcript levels of *HvLeg‐1* (Fig. 2a), *HvLeg‐5* (Fig. 2i) and *HvLeg‐7* (Fig. 2k) at 14 dpi as well as *HvLeg‐8* (Fig. 2m) at 7 dpi were up‐regulated compared to uninfected plants. However, mRNA levels of *HvLeg‐2* (Fig. 2c) and *HvLeg‐3* (Fig. 2e) declined in leaves of infected barley at 21 dpi.

### Barley cystatin *Icy‐4* gene expression changes on infection

The gene expression patterns of *Icy‐4* encoding HvCPI‐4 cystatin, the only barley cystatin with a long C‐terminal extension that can inhibit activity of VPEs, was determined to seek a possible regulatory mechanism of VPE activity in infected plants. A significant enhancement of *Icy‐4* gene expression was detected in leaves of infected plants at 7, 14 and 21 dpi (Fig. 2o). In infected roots a slight, but still statistically significant, reduction of *Icy‐4* mRNA content occurred only at 14 dpi (Fig. 2p).

### Live imaging of VPEs

**Figure 3:**
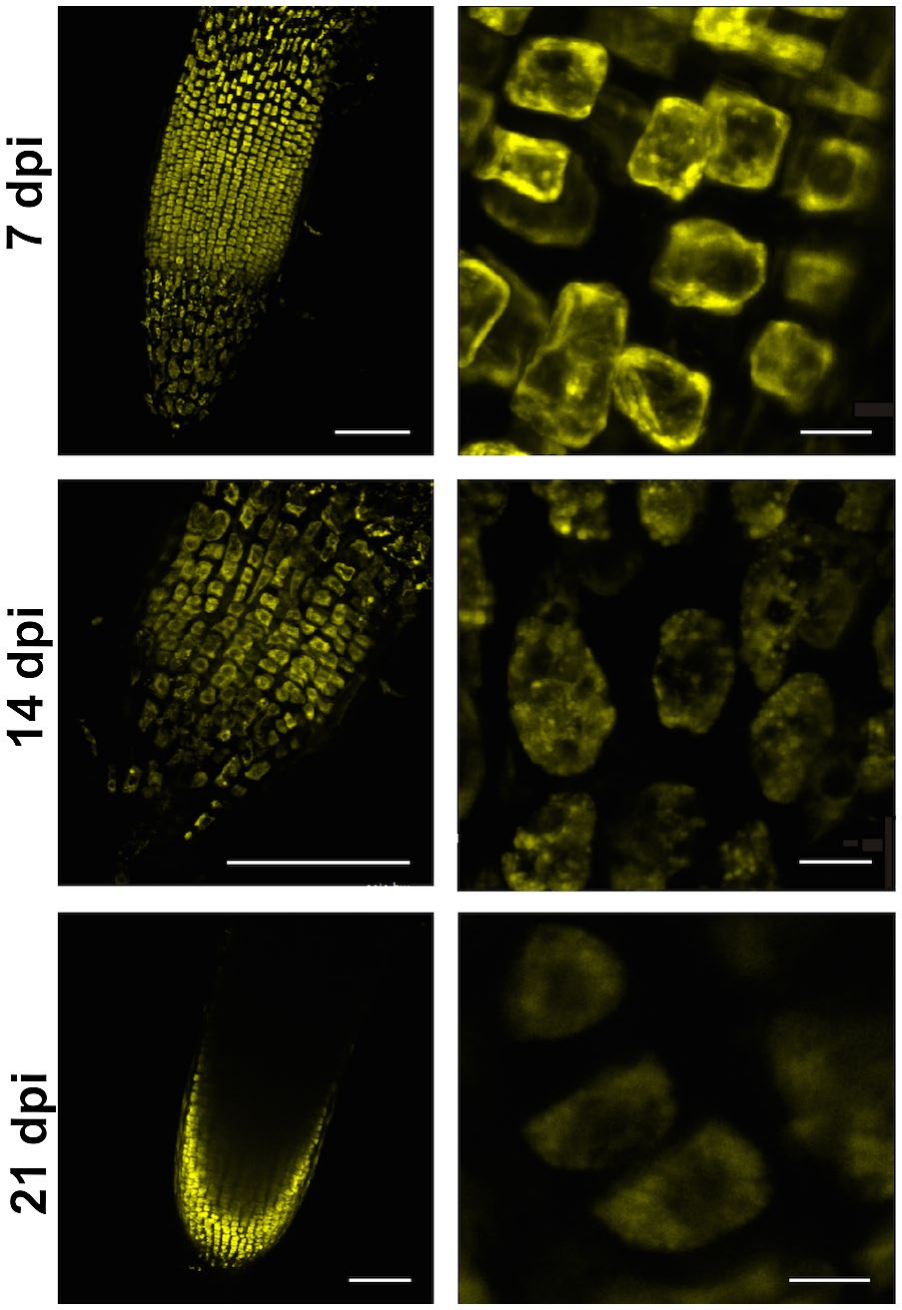
Vacuolar processing enzyme (VPE) localization in barley root tips observed under confocal laser scanning microscope at 7, 14 and 21 days post‐inoculation (dpi) with *Heterodera filipjevi*. Fluorescent staining with JOPD1 probe. VPE activity was localized in cells of the root elongation zone. Bar = 100 µm for left panel and 10 µm for right panel.

**Figure 4:**
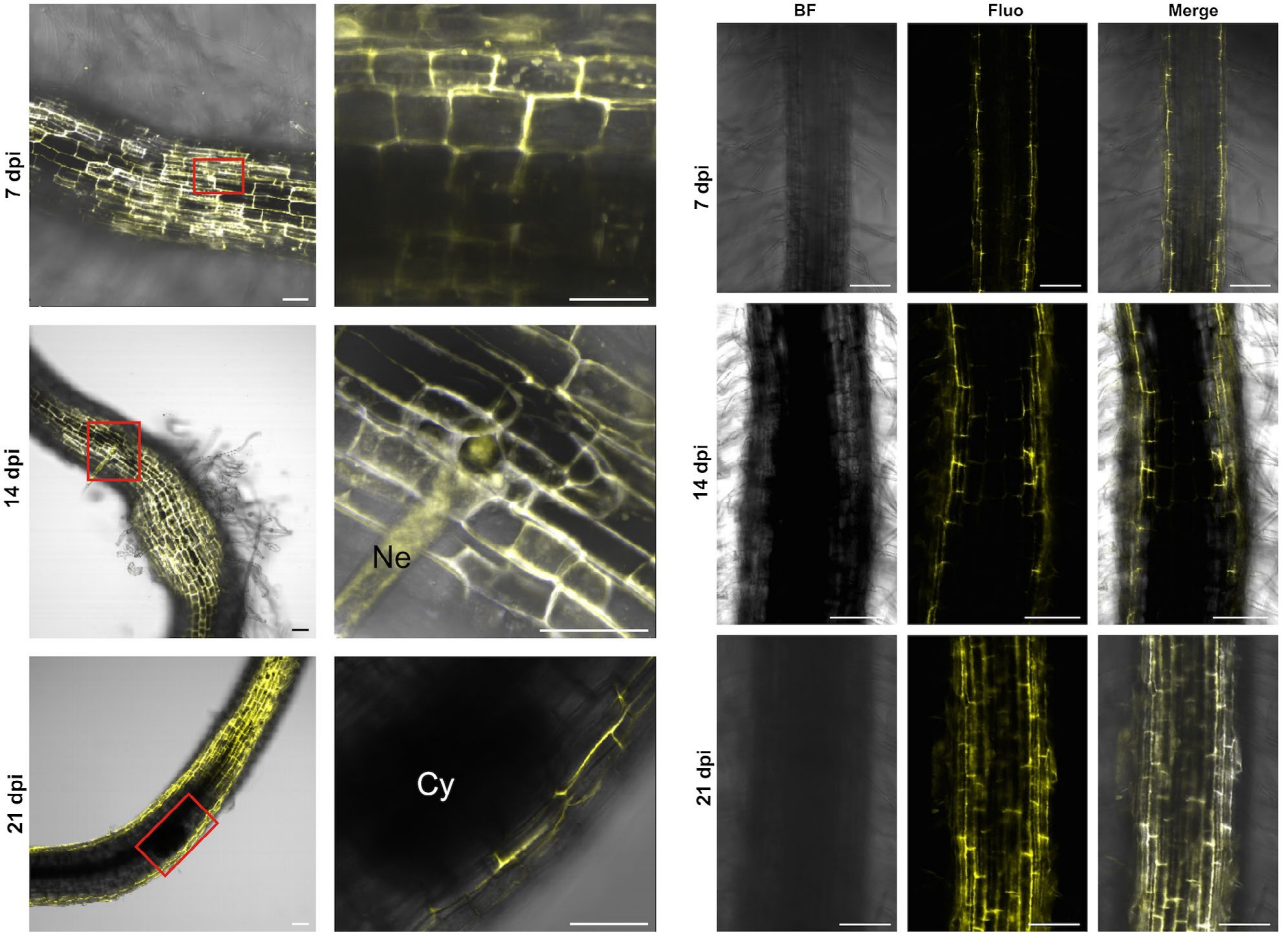
Left panel: vauolar processing enzyme (VPE) localization in barley roots observed under confocal laser scanning microscope at 7, 14 and 21 days post‐inoculation (dpi) with *Heterodera filipjevi*. Fluorescent staining with JOPD1 probe. Yellow signals were localized in vacuoles close to the nematode head in the infection zone (red rectangles). In the final period of observation, fluorescent signals were not detected at the infection site. Bar = 50 µm. Ne, nematode; Cy, cyst. Right panel: VPE localization in control uninfected barley roots. Bar = 100 µm. BF, bright field; Fluo, fluorescence.

Observations of VPE activity using a confocal laser scanning microscope revealed an intensive JOPD1 (a specific fluorescent probe for VPEs) fluorescent signal in the cells of the root apical meristem in both control and infested plants (Fig. 3). The infection did not change the fluorescent signal intensity in the root apical meristem region, therefore only the results after infection are presented and they were identical in control plants. As the left panel of Fig. 4 shows, in the roots containing syncytia at 7 and 14 dpi, a strong JOPD1 signal was recorded as numerous fluorescently labelled spherical structures exhibiting VPE activity. They were especially abundant next to the nematode’s head and in the apical regions of the expanding syncytium. At 21 dpi, VPE activity was almost unnoticeable within the whole syncytium, but it was visible in the root cells surrounding the syncytium (Fig. 4). For comparison, the results of observations of uninfected roots are showed in the right‐hand panels of Fig. 4.

### Cyclotide kalata B1 elicits methuosis in pre‐parasitic juveniles

Keeping in mind that legumains may produce cyclotides, we examined the effect of exogenous application of kalata B1 on the pre‐parasitic second‐stage juveniles of *H. filipjevi*. The exposition of larvae on different concentrations of kalata B1 resulted in a decrease in their mean survival time (Table 2). Statistical evaluation of obtained data and implementation of the mix effects Cox model showed that the control (without kalata B1) differed from all used concentrations. However, particular concentrations did not significantly differ from each other.

**Table 2:** Mean survival time of pre‐parasitic second stage juveniles of *Heterodera filipjevi* in different concentrations of the cyclotide kalata B1.

Kalata B1 concentration (µg ml^−1^)	Mean survival time	No. of nematodes in five drops	No. (proportion, %) of juveniles that survived till end of experiment
0	1083	40	30 (75)
50	566	68	23 (34)
100	552	59	18 (31)
250	469	53	14 (26)
500	620	63	24 (38)
750	490	76	22 (29)
1000	442	52	12 (23)

To elucidate why the pre‐parasitic juveniles die during incubation in kalata B1 solutions, we carried out a series of microscopic observations. In order to stimulate their parasitic behaviour, namely to induce stylet thrusting and head movements and thus allow uptake of tested molecules from the incubation medium to their intestine, we prepared an extract of fresh barley roots. Interestingly, the application of both the barley root extract (Video S1) and kalata B1 (Video S2) to a water drop in which juveniles were suspended resulted in head and stylet movements but the head and stylet movements on the kalata B1‐exposed nematodes were much slower and rarer than on the root extract‐exposed nematodes. The behaviour of the root extract‐exposed nematodes was analogous to that observed during the initial stages of root invasion when the juvenile migrates inside the root. This phenomenon did not occur when the juveniles were suspended in sterile water without any additives (Video S3).

**Figure 5:**
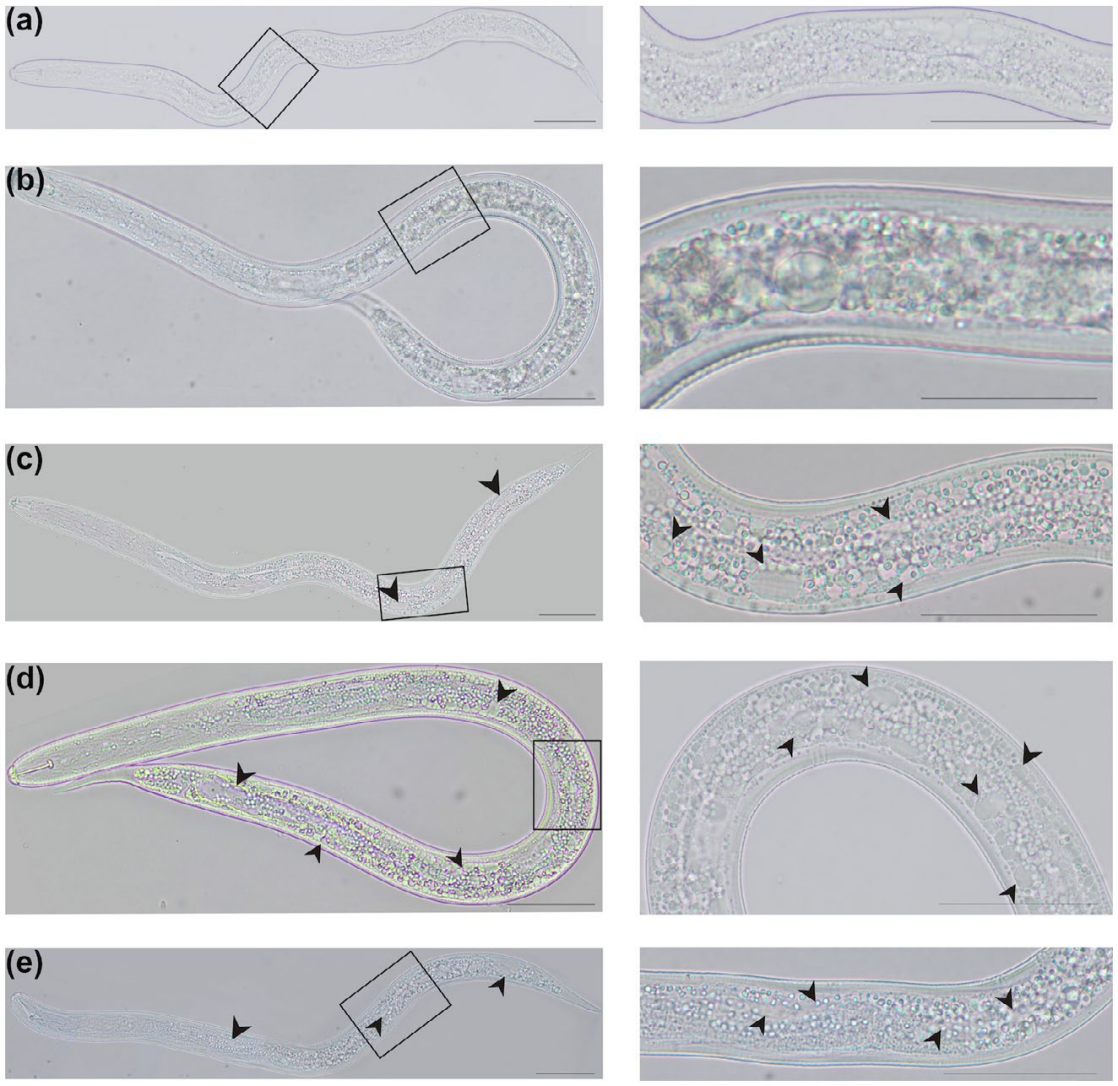
Influence of the cyclotide kalata B1 on vesiculation and methuosis in *Heterodera filipjevi* pre‐parasitic juveniles. Observations were performed under a light microscope. Giant voids and small granules (arrowheads) were observed in nematode bodies after incubation with different kalata B1 concentrations at 24 h. Incubation in (a) sterile water, (b) barley root extract, (c) 250 µg/mL kalata B1, (d) 750 µg/mL kalata B1 and (e) 1 mg/mL kalata B1. Bar = 50 µm.

Treatment of juveniles with kalata B1 solutions led to the formation and aggregation of numerous small granules in their intestines and the formation of large voids between them (Fig. 5). Finally, granules moved along with the liquid filling the intestine from the back of the body to the procorpus region and the continuity of the cuticle was broken between the stylet and metacorporal pump, leading to the release of granules into the external environment (Video S4). Such destruction was not observed in juveniles incubated in water or root extracts.

**Figure 6:**
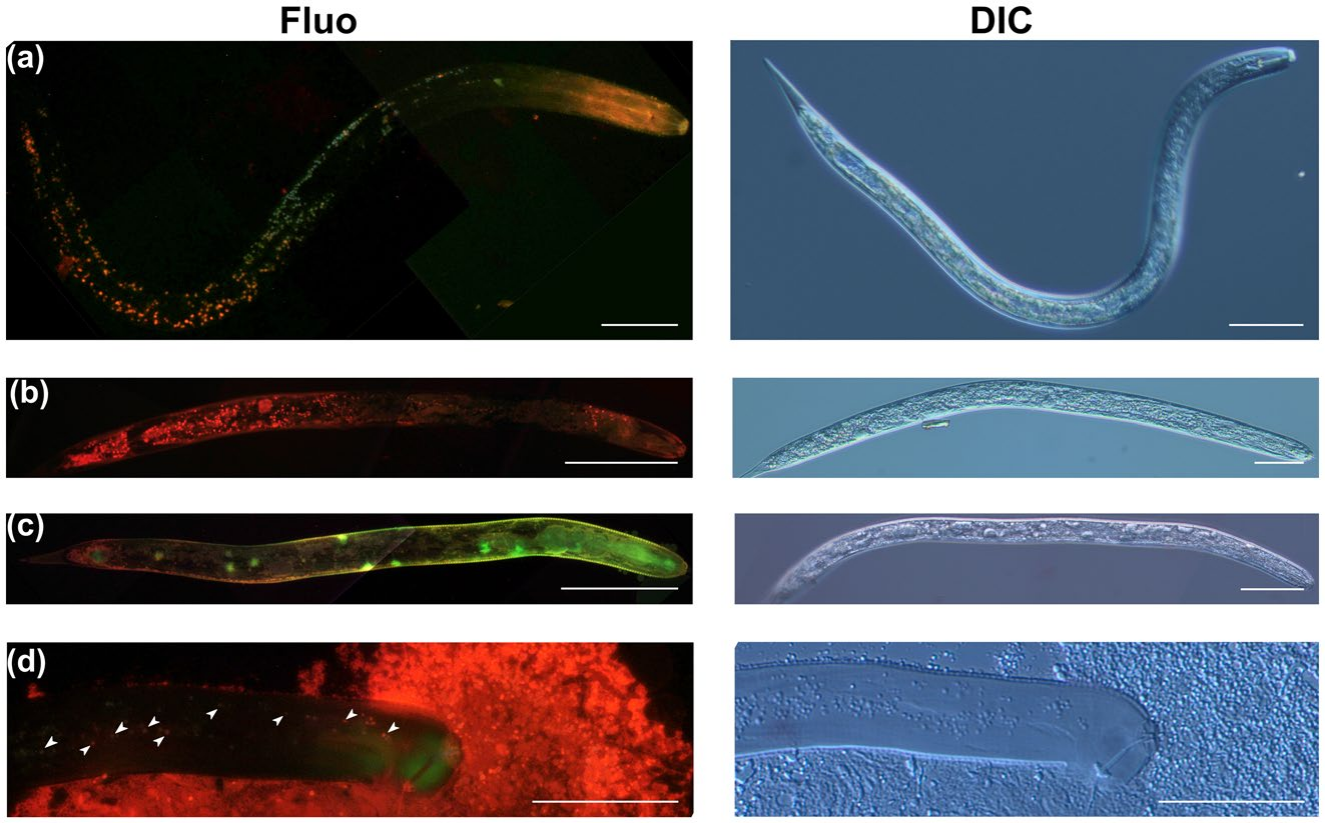
Left panel: neutral red (4 µM) staining of second‐stage pre‐parasitic juveniles of *Heterodera filipjevi* after 24 h incubation in (a) barley root extract and various concentrations of kalata B1: (b) 250 µg/mL and (c, d) 1 mg/mL. Observations were performed under an epifluorescence microscope. Arrowheads indicate granules stained by neutral red (d). Right panel: juvenile morphology observed under differential interference contrast (DIC) microscopy. Bar = 50 µm. Fluo, fluorescence.

In the next step, we added neutral red dye to incubation solution containing barley root extract or kalata B1 to investigate in more detail the properties of the granules induced by kalata B1. The neutral red dye is eurhodin dye, which shows different colouration depending on pH. In our study, neutral red penetrated the inside of the nematode body and the granules specifically stained red, indicating that their interior has an acidic pH (equal to pH 6.8 or below). Red‐stained granules were also occasionally present in the body of juveniles treated with root extract (Fig. 6a). This indicates that the low number of granules is not a pathological event. In addition, the number of granules increased dramatically after treatment with kalata B1, especially in the posterior part of the body, which can be considered as a pathological condition (Fig. 6b–d).

**Figure 7:**
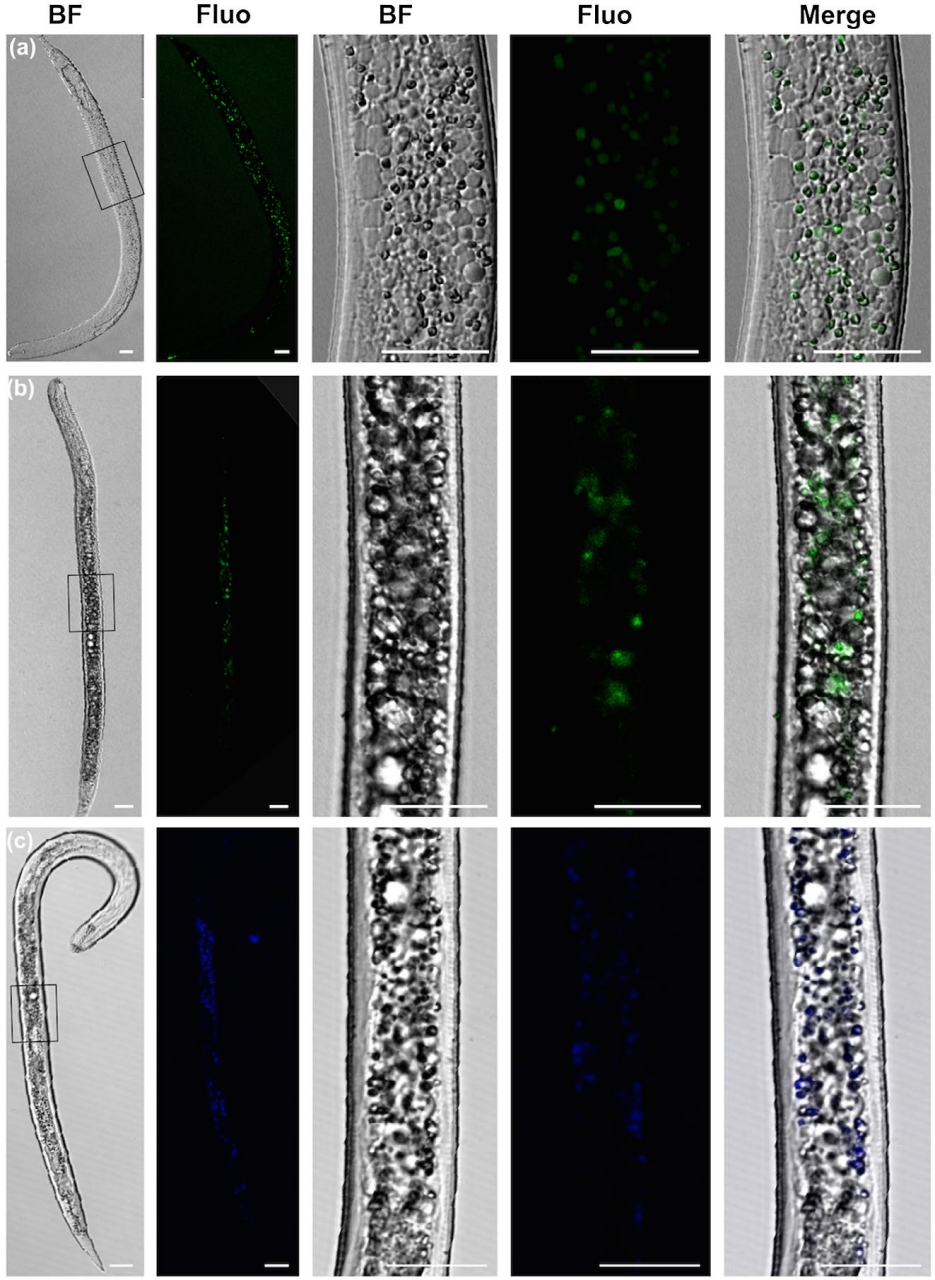
*In vivo* fluorescence labelling of reactive oxygen species (ROS) in *Heterodera filipjevi* larvae after 24 h incubation in 250 µg/mL kalata B1 observed under a confocal laser scanning microscope. Singlet Oxygen Sensor Green (a) detected singlet oxygen molecules in small granules; CellROX Green Reagent (b) specifically detected increased production of ROS in the mitochondria and nuclei; and CellROX Orange Reagent (c) revealed increased production of ROS in the cytoplasm. Bar = 20 µm. BF, bright field; Fluo, fluorescence.

In order to gain more background data concerning the mechanisms involved in the death of pre‐parasitic juveniles after cyclotide kalata B1 administration, we checked whether the production of reactive oxygen species (ROS), which play an important role in the progression of many disorders and diseases, is involved. We used three different fluorogenic probes to visualize the level of oxidative stress. First, we used Singlet Oxygen Sensor Green (SOSG) reagent to detect singlet oxygen molecules in nematodes treated with kalata B1. Regularly shaped, fluorescent, spherical structures were localized anteriorly to the above‐mentioned granules (Fig. 7a). Secondly, we used two other dyes to show the presence of ROS. The signals from CellROX Green reagent indicated an increase production of ROS in the mitochondria and nuclei (Fig. 7b), whereas CellROX Orange revealed enhanced ROS accumulation in the cytoplasm (Fig. 7c). Similarly to SOSG, the emitted fluorescence was largely associated with granules induced by kalata B1. Examples of negative controls for when the nematodes were incubated on water and then labelled with the ROS probe and when the ROS probe was omitted in the same experimental set‐up are presented in Fig. S1.

## Discussion

Cyst nematodes invade host roots and initiate formation of syncytia, which become the only source of nutrients for nematodes throughout their entire lifecycle. Here, in the *H. vulgare*–*H. filipjevi* pathosystem, we provide experimental evidence to elucidate the molecular mechanisms of plant and cyst nematode interaction.

Biotrophic parasites rely on the metabolism of invaded plants. For this reason, tissue damage in hosts must be limited until reproduction of nematodes has finished. The content and composition of photosynthetic pigments are effective markers of the physiological status of infected plants (Labudda *et al.*, 2018). Our results indicate that *H. filipjevi* causes only moderate alterations in content and composition of photosynthetic pigments in infected barley. This is because the nematode must ensure a continuous influx of photosynthetic nutrients to the syncytium, and thus keep the infected plant alive, relatively unchanged despite the stress caused by infection. Although the general physiological condition of the infected plant is not bad, for example the carotenoid content in leaves did not alter significantly, suggesting that non‐enzymatic antioxidant mechanisms and protection of the photosynthetic machinery work efficiently, our results clearly demonstrate the significant changes in the processes associated with the activity of proteolytic enzymes.

Eight genes encoding VPEs occur in the barley genome and they have different expression patterns in vegetative and generative organs (Julián *et al.*, 2013; Radchuk *et al.*, 2011, 2018). We analysed changes in the expression of seven genes during the course of infection. Expression of *HvLeg‐6* was not detected, but in general this gene is known to be expressed only in the immature zygotic embryo and nucellus (Bi *et al.*, 2005; Linnestad *et al.*, 1998). Together with the diverse expression of genes for VPEs, it was observed that TLA decreased during barley infection with *H. filipjevi.* Several molecular mechanisms may participate in this phenomenon. One of these mechanisms is the possibility that *H. filipjevi* inhibits VPE activity by effectors in a targeted way. Cyst nematodes produce many effectors, and it seems that two of them may be of particular importance in this process. Hütten (2018) identified the cystatin‐like (cysteine proteases inhibitor) effector in *H. schachtii*. It cannot be ruled out that the decreased TLA in roots of infected barley is to some extent caused by the action of cysteine protease inhibitors secreted by parasitizing juveniles. Our conviction that diminished TLA results from effectors secreted by *H. filipjevi,* rather than as result of plant regulatory mechanisms, is due to the fact that expression of the barley *Icy‐4* gene, coding for cystatin HvCPI‐4, was unchanged or down‐regulated in nematode‐infected roots. Another example of an effector that affects regulation of plant gene expression is the recently identified effector 30D08 from *H. schachtii*. This protein is secreted from the dorsal gland of juveniles and targeted to the plant nucleus, where it interacts with an auxiliary spliceosomal protein and thus affects splicing of pre‐mRNA and expression of a gene essential for syncytium formation (Verma *et al.*, 2018). The *β* *VPE* gene (AT1G62710.1) is among the genes in which expression is significantly changed by the *H. schachtii* 30D08 effector in syncytia induced in *A. thaliana* roots (Verma *et al.*, 2018).

Analysing the barley–*H. filipjevi* interaction, we have to focus on at least two basic issues. First, VPEs induce vacuolar rupture, which can lead to the activation of the proteolytic cascade and plant programmed cell death (PCD) during the response to pathogen infection (Hatsugai *et al.*, 2015). The plant tries to avoid the establishment of interaction with the biotrophic pathogen and the potential spread of the disease. It attempts to induce PCD in cells affected by the parasite, but as the cyst nematodes secrete the effectors inhibiting the gene expression and enzymatic activity of VPEs, PCD is limited or impeded and the infection process can process. This indicates that an obligate biotrophic pathogen, such as *H. filipjevi*, takes advantage of the inhibition of VPE activity in the infected host roots to survive and avoid their proteolytic defence responses. This assumption is also supported by data provided by Hütten *et al. *(2015), who showed a significant decrease in VPE activity in syncytia induced by *H. schachtii* in *A. thaliana* roots. However, the fact that we observed fluorescently labelled spherical structures with VPE activity close to the nematode’s head and in the apical region of the growing syncytia at 7 and 14 dpi cannot be ignored. Similar structures were observed by Baranowski *et al. *(2018) in syncytia induced by *H. schachtii* in *A. thaliana* roots. Some of these were considered to be lytic vacuoles and our results confirm these observations because the activity of legumains is inseparably associated with lytic vacuoles.

VPEs catalyse not only the peptide‐bond cleavage reaction but also production of defence proteins against pests and pathogens, including cyclotides (backbone‐cyclized proteins) (Hatsugai *et al.*, 2015). It turns out that the inhibited VPE activity promotes nematode infection through limited plant PCD (Siddique *et al.*, 2014) and decreased production of cyclotides that induce death of pre‐parasitic juveniles as shown here. Methuosis is a nonapoptotic cell death pathway whose name originates from the Greek *methuo*, meaning ‘to drink to intoxication’. The most visible feature in cells undergoing methuosis is the accumulation of fluid‐filled cytoplasmic vacuoles derived from macropinosomes (Maltese and Overmeyer, 2014). Similar formation of vacuoles in nematode bodies was recently described for *Meloidogyne incognita* exposed to oxalic acid (Jang *et al.*, 2016) and in *Bursaphelenchus xylophilus* incubated in 5‐iodoindole (Rajasekharan *et al.*, 2017). In both cases, morphological variations in the internal organs of treated nematodes and formation of vacuoles/granules and large voids within nematode bodies occurred, which are similar to the morphological alterations in *H. filipjevi* pre‐parasitic juveniles treated with kalata B1. We therefore propose that the increased mortality of the juveniles results from the progressive formation of numerous vacuoles/granules, which matches well with the methuosis phenotype.

It is well known that ROS are involved in many nematode physiological processes (Miranda‐Vizuete and Veal, 2016). At lower concentrations, they are involved in cell signalling, whereas at higher concentrations they can be toxic by induction of oxidative stress, leading to oxidative damage of the cell‐building molecules (Miranda‐Vizuete and Veal, 2016). Our experiments show that, apart from the induction of formation of numerous granules, an additional mechanism of kalata B1 toxicity is stimulation of ROS production. It can therefore be assumed that the increased production of ROS strengthens the toxic effect of cyclotides against nematodes, so oxidative damage in nematode bodies may occur, which results in shorter lifespan or death (Gems and Doonan, 2008).

Taken together, our results support the hypothesis that VPEs are important molecular players in plant–cyst nematode interactions and have a significant impact on the current knowledge regarding the molecular mechanisms of this biotrophic interaction. For the first time, the mechanism that lies at the basis of inhibited VPE activity in pathogen‐infected plants is explained. It has been proven that the cyst nematodes inhibit the activity of VPEs in the infected roots in a deliberate way, to prevent the negative effects that would influence their fitness and the development of feeding sites. We have shown that one potential VPE activity product, cyclotides, known polypeptides with strong antiparasitic properties, can lead to the death of juveniles by methuosis. Thus, we strongly suggest that the significantly reduced host VPE activity, probably through nematode effectors, promotes the infection and leads to avoidance of the activation of the proteolysis‐dependent plant defence response (including PCD) and also protects the invading juveniles from death by methuosis.

## Experimental Procedures

### Plant material and growth conditions

The fodder spring barley *Hordeum vulgare* ‘Airway’, characterized by high protein content in grains, was used. Seeds were rinsed in tap water for 2 h and then surface decontaminated in 5% (w/v) sodium hypochlorite solution enriched with 0.2% (v/v) Tween 20 for 10 min with gentle stirring. They were washed under tap water for 1 h and then incubated for 1 h on a magnetic stirrer in 0.2% (v/v) Plant Preservative Mixture (PPM) (Plant Cell Technologies, Inc., Washington DC, USA) to eliminate microbial contamination. The decontaminated seeds were placed (embryos upwards) side by side into Petri dishes (9 cm diameter) on a 0.2% (v/v) PPM‐soaked filter paper and covered with an identical one. After overnight incubation at 4 °C in the dark, the seeds were left in the dark at room temperature for another 2 days. Geminating seeds were transferred singly into a 50 mL Falcon‐type tube that was filled with sterilized (180 °C, 18 h) white quartz sand (0.5 mm granulation) (Pariyar *et al.*, 2010) and 15 mL of 0.2 × Knop medium (pH 6.4) was added to each tube. Plants were cultivated in a growth chamber MLR‐350 (Sanyo, Tokyo, Japan) at 23 ± 2 °C with a 16 h/8 h light/dark cycle under a photosynthetic photon flux density of 100 ± 25 μmol/m^2^/s and at 70–80% humidity. Every 4 days plants were watered with 8 mL sterile milli‐Q water.

### Nematode collection and inoculation

Cysts of *H. filipjevi* were sampled from naturally nematode‐infested experimental wheat fields of the International Maize and Wheat Improvement Center (CIMMYT) in Yozgat (39°08'N, 34°10'E; altitude 985 m a.s.l.) in the Central Anatolian Plateau of Turkey. Rhizospheres and roots of wheat plants were collected at the end of the growing season in 2013 and cysts were extracted by the modified flotation decanting method (Ashrafi *et al.*, 2017). Hatching of J2s was stimulated by soaking cysts in sterile 3 mM ZnCl_2_ at 17 °C. Freshly hatched juveniles were washed three times in sterile milli‐Q water. Tubes with 7‐day‐old barley plants were randomly divided into two groups, uninfected controls and infected ones. Plants from the latter were inoculated with approximately 200 freshly hatched J2s of *H. filipjevi* suspended in sterile milli‐Q water per tube. Infected plants were collected at 7, 14 and 21 dpi together with control uninfected plants (including 0 dpi). Six plants were used for each experimental bulked sample. Experiments were carried out in three independent biological replicates.

### Total RNA extraction and first‐strand cDNA synthesis by reverse transcription reactions

Total RNA was extracted from liquid nitrogen‐frozen powders of leaves and roots of uninfected and infected plants using the universal RNA purification kit (EURx, Gdańsk, Poland) according to the manufacturer’s instructions. During extraction, samples were treated with RNase‐free DNase I (EURx) directly on columns to digest contaminating genomic DNA. The RNA amount was measured spectrophotometrically (NanoDrop ND‐1000; Thermo Scientific, Waltham, MA, USA), and its purity and integrity were verified on 1.2% (w/v) agarose gel containing SimplySafe (EURx). Horizontal electrophoresis was run in 1 × TBE buffer (89 mM Tris, 89 mM boric acid, 2 mM EDTA, pH 8.3). Purified total RNA samples (2 μg) were reverse‐transcribed using a high‐capacity cDNA reverse transcription kit with MultiScribe Reverse Transcriptase and random primers (Applied Biosystems/Thermo Scientific).

### Gene expression profiling by quantitative reverse transcription‐PCR

Sequences of primers (Table S1) fors *HvLeg‐1* to *HvLeg‐7* , *TEF1 α‐subunit* and *actin* were adopted from Radchuk *et al. *(2011), *Icy4* from Martinez *et al. *(2009) and others were designed using OligoAnalyzer Tool (Integrated DNA Technologies, Skokie, Illinois, USA). *Hordeum vulgare translation elongation factor 1 α‐subunit* gene (*TEF1 α‐subunit*, accession no. Z50789.1), which presented stable expression over the whole period of infection experiment, was used as the endogenous reference gene. Quantitative reverse transcriptase‐PCR (qRT‐PCR) was conducted in Hard‐Shell 96‐well plates sealed with Microseal ‘B’ PCR films (Bio‐Rad, CA, USA) on a CFX96 Touch Real‐Time PCR Detection System (Bio‐Rad). Reaction mixtures contained 7.5 μL of PowerUP SYBR Green PCR Master Mix (Applied Biosystems/Thermo Scientific), 1.9 μL of sterile water, 200 nM primers and 5 μL of 1:25 diluted first‐strand cDNA template. qRT‐PCR conditions were as follows: AmpliTaq Gold polymerase (Applied Biosystems/Thermo Scientific) activation at 95 °C for 10 min, then 40 cycles consisting of denaturation at 95 °C for 15 s and annealing/extension at 60 °C for 60 s with fluorescence reading. Expression of each gene was tested in three biological replicates and three technical repetitions. The specificity of amplifications was tested by melting curve analysis. The reaction efficiency was calculated using LinRegPCR software (Ramakers *et al.*, 2003) whereas the absolute normalized gene expression levels and statistical significance of their differences were estimated by REST2009 software (Pfaffl *et al.*, 2002).

### Total legumain activity assay

Total legumain activity (TLA) was measured using the modified method described by Teper‐Bamnolker *et al. *(2017). Barley leaves and roots from infected and uninfected plants were frozen in liquid nitrogen and, without thawing, homogenized in liquid nitrogen with a mortar and pestle. The powder samples obtained (each about 100 mg) were extracted with 500 µL of ice‐cold extraction buffer (50 mM sodium acetate pH 5.5, 50 mM NaCl, 1 mM EDTA, 100 mM DTT, 1 mM PMSF). Tubes with samples were agitated (800 rpm, 4 °C, 15 min), centrifuged (16 100 ***g***, 4 °C, 30 min) and finally supernatants were collected.

Aliquots (20 μL) of the freshly prepared supernatants were mixed with 80 μL of 100 µM fluorogenic artificial substrate for legumains (Z‐Ala‐Ala‐Asn‐7‐amino‐4‐methylcoumarin; AMC) (Bachem, Bubendorf, Switzerland) dissolved in the activity assay buffer (50 mM sodium acetate pH 5.5, 50 mM NaCl and 4% (v/v) DMSO). Assays were conducted in black MaxiSorp 96‐well plates (Thermo Scientific) on a Varioskan LUX Multimode Microplate Reader (Thermo Scientific). Progress of the substrate turnover was followed at 37 °C at 380 nm excitation and 460 nm emission wavelengths for 60 min with fluorescence reads every 5 min. Linear progression of the enzymatic reaction was checked and blank samples considering fluorescence of the substrate and plant extracts were prepared as well. The total legumain activity was expressed as pmol of released 7‐amino‐4‐methylcoumarin (AMC) after 1 min per gram of fresh weight (FW). The AMC concentration was determined from a standard curve. The reaction conditions and the specificity of the method used were checked by the activity measurement of recombinant human legumain/asparaginyl endopeptidase (catalogue no. 2199‐CY‐010, R&D Systems, Minneapolis, MN, USA).

### Pigments content assay

The concentrations of photosynthetic pigments were determined according to the method of Lichtenthaler (1987). Leaf samples (50 mg) were homogenized in 5.0 mL of ice‐cold 80% (v/v) acetone and centrifuged for 15 min at 4 °C (2500 ***g***). The absorbance of acetonic extracts was measured at 470, 646 and 663 nm. The chlorophyll *a* (chl *a*), chlorophyll *b* (chl *b*) and carotenoid concentrations were calculated according to Wellburn (1994). Total chlorophylls (chl *a* + *b*), the ratio of chlorophyll *a* to *b* (chl *a*:*b*) and the ratio of total chlorophylls to carotenoids (chl *a* + *b*:carotenoids) were also calculated.

### *In vivo* labelling of VPEs

Control and nematode‐infected roots of barley plants were incubated in 70 mM sodium acetate buffer (pH 5.5) containing 100 mM DTT and 1.6 µM JOPD1 (a specific fluorescent probe for VPEs) for 2 h at room temperature in the dark with gently agitation. Unlabelled roots were used as a control. After incubation, roots were washed three times with 50 mM sodium acetate buffer (pH 5.5) and examined under a Leica TCS SP5II confocal laser scanning microscope (Leica Microsystems, Wetzlar, Germany) with excitation and emission wavelengths set at 514 nm and 560–580 nm, respectively.

### Morphology of *H. filipjevi* juveniles

To assess morphology and the survival of nematodes after treatment with kalata B1, the nematodes were suspended in water drops supplemented with kalata B1 (50, 100, 250, 500, 750 and 1000 µg/mL) and pure water as a control. Five water drops (50 µL) containing juveniles were used for observations under bright field and differential interference contrast (DIC) microscopy. Observations were performed at the beginning of experiment and after 0.5, 1.0, 1.5, 2.5, 4.0 and 24 h. Examinations of neutral red staining (4 µM) were performed under an Olympus AX70 Provis epifluorescence microscope equipped with a UM61002 fluorescence filter set and an Olympus UC90 digital camera (Olympus, Tokyo, Japan). Studies of juvenile morphology and changes in their behaviour in response to root homogenates (200 mg roots homogenized in 1 mL water and centrifuging for 10 min at 16 000 ***g***) were also performed on the same microscope.

### ROS detection

Second‐stage juveniles of *H. filipjevi* were incubated for 24 h in milli‐Q water or in 250 µg/mL solution of kalata B1 (XXC; ZZHp; ZZi; BBMm, >95% purity) commercially synthetized by bioWORLD (Dublin, OH, USA). ROS accumulation was observed under a Leica TCS SP5II confocal laser scanning microscope using CellROX Orange, Green Oxidative Stress Reagents or Singlet Oxygen Sensor Green (SOSG) (Life Technologies, Eugene, OR, USA) fluorescent probes according to the manufacturer’s instructions and Muszyńska *et al. *(2019). The excitation/emission spectra were 514/573–664, 488/500–574 and 504/525–598 nm, respectively.

Digital pictures were merged (if needed) using Photoshop CS6 (Adobe Systems Inc., San Jose, CA, USA) software (panorama tool), and adjusted using the same software by non‐destructive tools (contrast, levels and/or curves). All adjustments were made on the whole area of the picture.

### Statistical analysis

The results of enzyme activity and pigment content were subjected to one‐way ANOVA analysis. The significant differences between means were determined using Tukey’s test at *P* < 0.05. Statistica v. 13.3 was used to carry out the calculations. To analyse whether kalata B1 affects nematode survival, a Cox model with mixed effects (Ripatti and Palmgren, 2000; Therneau *et al.*, 2003) and the coxme package (Therneau, 2018) or R (R Core Team, 2019) were applied. The comparisons between particular concentrations were conducted through testing the corresponding linear hypotheses. Random effects were needed to take into account the nesting of nematodes within drops. After exploratory analysis, kalata B1 concentrations were treated as a qualitative factor. The significance level of 0.05 was used for the analyses.

## Supporting information


**Fig. S1** *In vivo* fluorescence labelling of reactive oxygen species (ROS) in *Heterodera filipjevi *larvae after 24 h incubation in 250 µg ml^−1^ kalata B1 observed under confocal laser scanning microscope. Figure presents example and representative results of control reactions for Figure 7. Left panel: the absence of fluorescence probe (Singlet Oxygen Sensor Green) was replaced by sterile water. Right panel: nematodes were incubated on water and then labelled with the ROS probe. Bar 50 = µm.* BF* bright field, *Fluo *fluorescence.

**Video S1** Effect of barley root extract on *Heterodera filipjevi* behaviour after 5 min exposure at room temperature.

**Video S2** Effect of cyclotide kalata B1 (250 µg ml^−1^) on *Heterodera filipjevi* behaviour after 5 min exposure at room temperature.

**Video S3** Effect of sterile water on *Heterodera filipjevi* behaviour after 5 min incubation at room temperature.

**Video S4** Death of *Heterodera filipjevi* by breaking the cuticle after 24 h incubation in 250 µg ml^−1^ cyclotide kalata B1 at room temperature.

**Table S1** Genes and primer sequences used in quantitative reverse transcription‐PCR analyses.
