# Supplementary material for: Activity profiling of barley vacuolar processing enzymes provides new insights into the plant and cyst nematode interaction
Source: Mol Plant Pathol. 2019 Oct 12;21(1):38–52. doi: 10.1111/mpp.12878 (PMC6913211; doi:10.1111/mpp.12878)
Supplement: Supplementary file 6 — Table S1 Genes and primer sequences used in quantitative reverse transcription‐PCR analyses. [file MPP-21-38-s006.docx]

| **Gene/alternative name** | **GenBank ID** |  | **Product size** |
| --- | --- | --- | --- |
| *HvLeg-1*  *(HvVPE1)* | AM941111.1 | 5′-CCGAAAGGCTGCAATCAACCAA-3′  5′-GCAGCTCGTACCTCCTCCACAAGA-3′ | 65 |
| *HvLeg-2*  *(HvVPE2b)* | AM941112.1 | 5′-CAGCGCTTGCAACGGCTACGA-3′  5′-TGCAAGCGGATCAGGGCTGTG-3′ | 72 |
| *HvLeg-3*  *(HvVPE2d)* | AM941113.1 | 5′-GCTGCCTTTGCCCATCCTG-3′  5′-TCCCCCGTTTAACTGCTCATACTT-3′ | 91 |
| *HvLeg-4*  *(HvVPE3)* | AM941114.1 | 5′-TGCTTGAACGAGCCTTAGGTGAAT-3′  5′-ATATTTGTTGCCGGGGAGAGTTCT-3′ | 115 |
| *HvLeg-5*  *(HvVPE4)* | AM941115.1 | 5′-CGGCCCGGACGACCACATC-3′  5′-CCCGCCGGCGTGCTTCTT-3′ | 133 |
| *HvLeg-6*  *(HvVPE2a)* | FR696361.1 | 5′-TGCGCTGCAGTACACGGAA-3′  5′-TCTAGCTAGCTAGGAACCTCCG-3′ | 57 |
| *HvLeg-7*  *(HvVPE2c)* | FR696363.1 | 5′-GCGTCTCTGAGGCCCAAATGA-3′  5′-TTATAACCGCCGCAAGCACTGAT-3′ | 51 |
| *HvLeg-8* | AK364023.1 | 5’-ACTCACTGCCATCCTTCTCAAGAGCTGT-3’  5’-AGACTTGAGAACCATTGGACCATCGGCA-3’ | 221 |
| *Icy4* | AJ748344.1 | 5’-TTGTTGAATGCGGCACGA -3’  5’-GCAGCCAACAGTACCCTGAGTT-3’ | 111 |
| *TEF1 α-subunit* | Z50789.1 | 5′-GACCGCCGCCGCCTCAT-3′  5′-GATACCAGCCTCAAAACCACCAGT-3′ | 448 |
| *Actin* | AY145451 | 5′-GGTAGGGATGGGGCAGAAGG-3′  5′-ACCAGCGAGATCCAAACGAAGAA-3′ | 415 |

Table S1. Genes and primer sequences used in quantitative reverse transcription‐PCR analyses.
